# Human tenocytes are stimulated to proliferate by acetylcholine through an EGFR signalling pathway

**DOI:** 10.1007/s00441-012-1530-5

**Published:** 2012-12-05

**Authors:** Gloria Fong, Ludvig J. Backman, Gustav Andersson, Alexander Scott, Patrik Danielson

**Affiliations:** 1 Department of Integrative Medical Biology, Anatomy, Umeå University, SE-901 87 Umeå, Sweden; 2 Department of Physical Therapy, University of British Columbia, Vancouver, BC Canada; 3 Centre for Hip Health and Mobility, Vancouver Coastal Health and Research Institute, Vancouver, BC Canada; 4 Department of Surgical and Perioperative Sciences, Sports Medicine, Umeå University, Umeå, Sweden

**Keywords:** Muscarinic acetylcholine receptors, Tendinopathy, Tendinosis, Choline acetyltransferase, Vesicular acetylcholine transporter, Atropine, Non-neuronal acetylcholine, Human

## Abstract

Studies of human patellar and Achilles tendons have shown that primary tendon fibroblasts (tenocytes) not only have the capacity to produce acetylcholine (ACh) but also express muscarinic ACh receptors (mAChRs) through which ACh can exert its effects. In patients with tendinopathy (chronic tendon pain) with tendinosis, the tendon tissue is characterised by hypercellularity and angiogenesis, both of which might be influenced by ACh. In this study, we have tested the hypothesis that ACh increases the proliferation rate of tenocytes through mAChR stimulation and have examined whether this mechanism operates via the extracellular activation of the epidermal growth factor receptor (EGFR), as shown in other fibroblastic cells. By use of primary human tendon cell cultures, we identified cells expressing vimentin, tenomodulin and scleraxis and found that these cells also contained enzymes related to ACh synthesis and release (choline acetyltransferase and vesicular acetylcholine transporter). The cells furthermore expressed mAChRs of several subtypes. Exogenously administered ACh stimulated proliferation and increased the viability of tenocytes in vitro. When the cells were exposed to atropine (an mAChR antagonist) or the EGFR inhibitor AG1478, the proliferative effect of ACh decreased. Western blot revealed increased phosphorylation, after ACh stimulation, for both EGFR and the extracellular-signal-regulated kinases 1 and 2. Given that tenocytes have been shown to produce ACh and express mAChRs, this study provides evidence of a possible autocrine loop that might contribute to the hypercellularity seen in tendinosis tendon tissue.

## Introduction

In recent years, increasing attention has been devoted to the non-neuronal expression of signal substances traditionally associated with neurons and the potential roles of such substances in various pathological conditions. Particular interest has been given to acetylcholine (ACh; Wessler et al. 2001), a classical neurotransmitter well-known as being localised in the central and peripheral nervous systems. Contemporary research has demonstrated that ACh and/or its synthesising enzyme, choline acetyltransferase (ChAT), are present in non-neuronal human cells, such as the epithelium of the airways and epidermis, endothelial and muscle cells and various immune cells (Wessler et al. 2001; Kawashima and Fujii 2003). A physiological role of non-neuronal ACh might explain the widespread presence of ACh receptors, such as muscarinic receptors (mAChRs), in tissues not innervated by cholinergic neurons (Wessler et al. 1998). Recently, we have demonstrated that mAChRs of subtype M_2_ (M_2_Rs) are expressed in blood vessels, nerves and primary fibroblastic cells (tenocytes) in both Achilles (Bjur et al. 2008) and patellar (Danielson et al. 2006) tendons of man, whereas cholinergic innervation is scarce or absent. The M_2_Rs are particularly abundant in tendons afflicted by tendinosis (Danielson et al. 2006, 2007; Bjur et al. 2008). Tendinosis frequently underlies tendinopathy, a clinical condition defined by chronic tendon pain and impaired function, and is characterised by a series of histopathological tissue changes (Khan et al. 1999), including tenocyte proliferation and angiogenesis. The above observations have led to the hypothesis that ACh plays a role in the development of tendinosis, since the stimulation of ACh receptors in other tissues has previously been shown to stimulate angiogenesis (Jacobi et al. 2002), modulate peripheral nociception (Vogelsang et al. 1995; Wess et al. 2003) and promote the proliferation of liver myofibroblasts (Oben et al. 2003). Furthermore, tenocytes in vivo have been shown to produce a variety of signal substances traditionally associated with neurons, especially in tendinosis (for a review, see Danielson 2009). A striking finding in this regard is the suggested increased production and release of ACh in tendinosis tissue, as evidenced by a higher expression of ChAT and the vesicular ACh transporter (VAChT) in tenocytes (Danielson et al. 2006, 2007; Bjur et al. 2008).

As is well-known, G-protein coupled receptors (GPCRs), to which mAChRs belong, can transduce mitogenic signals leading to proliferation (New and Wong 2007) and evidence has suggested that an ACh stimulus can result in the proliferation of fibroblast-like cells via these receptors (Matthiesen et al. 2006, 2007). In fibroblasts, proliferation is typically mediated by classical mitogen-activated protein kinases (MAPK), namely extracellular-signal-regulated kinases 1 and 2 (ERK1/2; Matthiesen et al. 2007). As has also been shown in a variety of cell types, the stimulation of GPCR can result in the transactivation of the epidermal growth factor receptor (EGFR), leading to the activation of ERK1/2 (New and Wong 2007; Xie et al. 2009). In some cells, the GPCR-EGFR transmission appears to be independent of extracellular ligands (relying on cytoplasmic mechanisms), whereas in other cell types, cell-surface-bound pro-ligands are processed and released to the extracellular matrix by metalloproteinases (MMPs; Kraus et al. 2003; Rozengurt 2007). These ligands subsequently bind to and stimulate the EGFR.

The role that mAChRs might play in the modulation of human tendon cell behaviour is unknown. Furthermore, whether primary Achilles tendon cell cultures maintain their expression of mAChRs in vitro or retain their capacity for ACh production is also unknown. Therefore, in this study, we have examined the presence of mAChRs and the expression of the synthesising enzyme ChAT and of VAChT in primary Achilles tendon cell cultures. Moreover, given that tenocyte proliferation is a prominent and early feature of tendinosis and that the effect of ACh on tenocyte proliferation has not previously been studied, we have also examined the possible proliferative effect of ACh on tenocytes and its mechanism of action.

## Materials and methods

### Ethics statement

These studies were approved by the Regional Ethical Review Board in Umeå and were performed according to the principles of the Declaration of Helsinki. Written informed consent was received from all participants.

### Human tendon cell culture

Biopsies were isolated and cultured as previously described (Backman et al. 2011). Briefly, tendon tissue biopsies from the lateral mid-portion of the Achilles tendon of healthy donors were enzymatically digested and the cells were cultured in D-MEM supplemented with 10% fetal bovine serum (FBS; Invitrogen, Grand Island, N.Y., USA; code no. 16000), 1% pen-strep (Invitrogen; code no. 15140) and 0.2% L-glutamine (Invitrogen; code no. 25030) at 37°C in a humidified atmosphere of 5% CO_2_.

### Experimental conditions

The cells used in this study were in passages 3–6 to ensure a similar phenotype. All experiments were performed under serum-starved condition of 1%, a condition in which the cells had a healthy appearance and a steady proliferation rate. The serum-starved condition was initiated 24 h before the start of an experiment.

### Cell-signalling experiments

The cells were seeded in cell culture plates (BD Bioscience; BD Falcon, Bedford, Mass., USA; code no. 353004) at a density of 275,000 cells/plate and incubated under serum-starved conditions as described under “Experimental conditions”. The cells were then pre-treated with either the EGFR inhibitor, AG1478 (0.8 μM; Calbiochem, Darmstadt, Germany; code no. 658548), the metalloproteinase inhibitor, GM6001 (8 μM; Calbiochem; code no. 364206) or the muscarinic receptor antagonist, atropine (10^−5^ M; Sigma, Saint Louis, Mo., USA; code no. A0132) for 30 min. Following this, the cells were stimulated with ACh (10^−6^ M; Sigma; code no. A2661) or the vehicle (dimethylsuphoxide) for an additional 30 min. A further control without treatment was also included. To determine the levels of phosphorylated (phospho-)ERK1/2 and phospho-EGFR, Western blot analysis was performed (for details, see below)*.*

### Immunocytochemistry

Antibodies directed towards ChAT (1:50; Chemico, Temecula, Calif., USA; code no AB144P), M_2_R (1:100; Chemico; code no. AB5166), M_3_R (1:50; Novus Biologiocals, Littleton, Colo., USA; code no. NLS5259), M_4_R (1:50; Novus Biologiocals; code no. NLS220), M_5_R (1:50; Novus Biologiocals; code no. NLS1334), VAChT (1:25; Santa Cruz, Calif., USA; code no. sc-7716), tenomodulin (1:100; Santa Cruz; code no. sc-49325), vimentin (1:100; Dako, Glostrup, Denmark; code no. M0725), collagen I (1:100; Abcam, Cambridge, UK; code no. ab34710), collagen III (1:100; Abcam; code no. ab7778), scleraxis (1:25; Abcam; code no. ab58655), alpha-smooth muscle actin (1:100; Abcam; code no. ab5694) or connexin 43 (1:25; Santa Cruz; code no. sc-59949) were used. Cells (1.5 × 10^4^/well) were seeded on 8-well chamber slides (BD Falcon; code no. 354118) and allowed to adhere overnight before being fixed for 5 min in 2% paraformaldehyde in 0.1 M phosphate buffer (pH 7.4) and subsequently washed four times in phosphate-buffered saline (PBS) before being blocked in swine (for primary polyclonal antibodies raised in rabbit, i.e., the antibodies towards M_2_R, M_3_R, M_4_R, M_5_R, alpha-smooth muscle actin, scleraxis, collagen I and collagen III), donkey (for polyclonal goat-antibodies, i.e., antibodies towards ChAT, VAChT and tenomodulin) or rabbit (for the monoclonal mouse-antibody towards connexin 43) serum for 15 min. This was followed by incubation with the primary antibody for 60 min at 37°C or at 4°C overnight. After being washed four times, an additional blocking step was performed before incubation with the secondary antibody, a tetramethylrhodamine isothiocyanate (TRITC)-conjugated swine anti-rabbit IgG (Dako; Copenhagen, Denmark; code no. R0156), fluorescein isothiocyanate (FITC)-conjugated donkey anti-goat IgG (Jackson Immunoresearch; West Grove, Pa., USA; code no. 705-095-147) or a TRITC-conjugated rabbit anti-mouse (Dako; code no. R0270) for 30 min at 37°C. When double-staining with vimentin or tenomodulin was carried out, incubation with the secondary antibody was followed by four washes in PBS before a blocking step in normal rabbit or donkey serum for 15 min. The primary antibody was incubated with the cells for 60 min at 37°C, followed by an additional four washes in PBS and a blocking step in normal (rabbit, donkey) serum for 15 min. After incubation with the secondary antibody, namely a TRITC-conjugated rabbit anti-mouse IgG (Dako; code no. R0270) or FITC-conjugated donkey anti-goat IgG (Jackson Immunoresearch; code no. 705-095-147), the cells were washed four times in PBS and mounted with Vectashield Hard Set Medium with 4,6-diamidino-2-phenylindole (DAPI; Vector Laboratories; Burlingame, Calif., USA; code no. H-1500).

Expression of M_4_R in tenocytes was also confirmed on tissue sections from biopsies of normal healthy tendon tissue by using immunohistochemistry according to a previously used protocol (Bjur et al. 2008).

Control staining was performed by the substitution of the primary antibody with PBS. A Zeiss Axioskop 2 Plus microscope equipped for epifluorescence and an Olympus DP70 digital camera was used for analysis.

### Western blot

Cells were lysed in a buffer consisting of 150 mM sodium chloride, 1% Triton, 0.5% sodium deoxycholate, 0.1% sodium dodecyl sulphate (SDS), 50 mM TRIS, pH 8.0, freshly supplemented with a protease inhibitor cocktail at a dilution of 1:200 (Sigma; code no. P1860). Protein concentration was determined by using the Protein Assay Dye Reagent Concentrate (Bio-Rad, Hercules, Calif., USA; code no. 500–0006) with bovine serum albumin (BSA; Sigma; code no. A9647) as a standard. Total protein (20–40 μg) from each sample or the M_2_R peptide, used as a positive control (Chemico; code no. Ab5166), was diluted in a Laemmli sample buffer (Bio-Rad; code no. 161–0737) and boiled for 5 min before being loaded and separated on an SDS-polyacrylamide gel and then transferred to a polyvinylidene difluoride membrane (Santa Cruz; code no. sc-3723). Ponceau S staining (0.1% Ponceau red, 1% acetic acid diluted in MilliQ water) confirmed successful transfer before the staining procedure. Membranes were blocked in 5% non-fat milk at room temperature for 1 h before incubation with the primary antibodies. The antibody towards phospho-ERK1/2 (Thr202, Tyr204) was used at a dilution of 1:2000 (Cell signal, Danvers, Mass., USA; code no. 4370). According to the manufacturer, this antibody does not cross-react with the phosphorylation residues of JNK/SAPK or p38 MAP kinases. The antibody towards phospho-EGFR was used at a dilution of 1:1000 (Cell signal; code no. 3777) and, according to the manufacturer, it might weakly cross-react with other tyrosine-phosphorylated proteins. The antibody towards M_2_R, which is reported by the manufacturer not to exhibit any cross-reactivity with other muscarinic receptors, was used at a dilution of 1:1000 (Chemico; code no. AB5166). The antibody towards M_4_R was used at a dilution of 1:500 (Santa Cruz; code no. sc-9109), the antibody towards scleraxis at a dilution of 1:1000 (Abcam; code no. ab58655) and the antibody towards beta (β)-actin at a dilution of 1:2000 (Cell Signal; code no. 4967). All primary antibodies were raised in rabbit and immunostaining was performed overnight at 4°C. The membranes were then washed and incubated with horseradish peroxidase (HRP)-conjugated goat anti-rabbit secondary antibody, at a dilution of 1:2000 (Cell Signal; code no. 7074) for 1 h at room temperature. Band detection was performed by using chemiluminescent HRP substrate (GE Healthcare, Little Chalfont, Buckinghamshire, UK; code no. RPN2132) for 5 min prior to visualisation on high performance chemiluminescence film (GE Healthcare; code no. 28-9068-38).

To confirm equal protein loading, the membranes were stripped with a Western blot stripping buffer (Thermo Scientific, Rockford, Ill., USA; code no. 21059) and re-probed for β-actin expression.

### Cell viability

Crystal violet dye was used to measure cell viability as previously described (Backman et al. 2011). Briefly, 1.5×10^5^ cells/well were seeded in a 6-well plate and allowed to adhere overnight before serum starvation. Cells were washed in PBS to remove non-adherent cells prior to fixation in 1% glutaraldehyde for 30 min. After additional washes in PBS, the adherent cells were stained with 0.1% crystal violet (Sigma; code no. C3886), washed in water, air-dried and then permeabilised in 30% methanol and 10% acetic acid. The absorbance was read at 590 nm in a 96-well plate format. Experiments were performed in triplicate.

### Cell proliferation assay with 5-bromo-2′-deoxy-uridine

The manufacturer’s instructions were followed (Roche Applied Science, Mannheim, Germany; code no. 11299964001). Briefly, 1.5×10^4^ cells/well on an 8-well chamber slide (BD Falcon; code no. 354118) were pre-labelled with 5-bromo-2′-deoxy-uridine (BrdU) for 120 min at 37°C in a humidified atmosphere of 5% CO_2_. This was followed by fixation in a fixative solution (250 mM glycine, 100 ml ethanol, pH 2.0) for 20 min at 20°C. Following fixation, the cells were covered with a monoclonal BrdU antibody for 30 min at 37°C, before being incubated with anti-mouse-Ig towards alkaline phosphate for an additional 30 min at 37°C. Finally, the cells were covered in 4.3 mM nitroblue tetrazolium and 3.3 mM 5-bromo-4-chloro-3-indolyl-phosphate diluted in substrate solution (100 mM TRIS-HCl, 100 mM NaCl, 50 mM MgCl_2_, pH 9.5) and then mounted in Vectashield HardSet Medium with DAPI (Vector Laboratories; code no. H-1500). The percentage of BrdU-positive cells was determined. Experiments were performed in triplicate.

### Reverse transcription with quantitative polymerase chain reaction

As previously described (Backman et al. 2011), total RNA was isolated by using an RNeasy kit (Qiagen, Hilden, Germany; code no. 74106) and then 1 μg of this RNA was reversed-transcribed into cDNA by using a High Capacity cDNA Reverse Transcription kit (Applied Biosystems [ABI], Warrington, Cheshire, UK; code no. 4368813). Quantitative polymerase chain reaction (qPCR) was performed by using a TaqMan fast universal PCR master mix (ABI; code no. 4352042) and probes for M_2_R (ABI; code no. Hs00265208), M_3_R (ABI; code no. Hs00265216), M_4_R (ABI; code no. Hs00265219), M_5_R (ABI; code no. Hs00255278) and scleraxis (ABI; code no. Hs03054634). Each sample, run in duplicate, contained the probe (20×), master mix (2×), 20 ng sample and nuclease-free water to give a final volume of 10 μl for each reaction. Amplification was conducted under the following conditions: after 20 s of denaturation at 95°C, the amplification was carried out for 1 s at 95°C for denaturation and 20 s at 60°C for annealing/extension in an ViiA7 instrument. The expression level was determined relative to the endogenous control, β-actin (ABI; code no. 4333762F).

### Statistics

Data were statistically analysed by using PASW Statistics 18 (18.0.0; SPSS, Chicago, Ill., USA). One-way analysis of variance (ANOVA), followed by the Bonferroni post-hoc test, was applied. All results were successfully reproduced at least once. Significance was predetermined at *P*<0.05.

## Results

### Phenotyping of cells

**Fig. 1:**
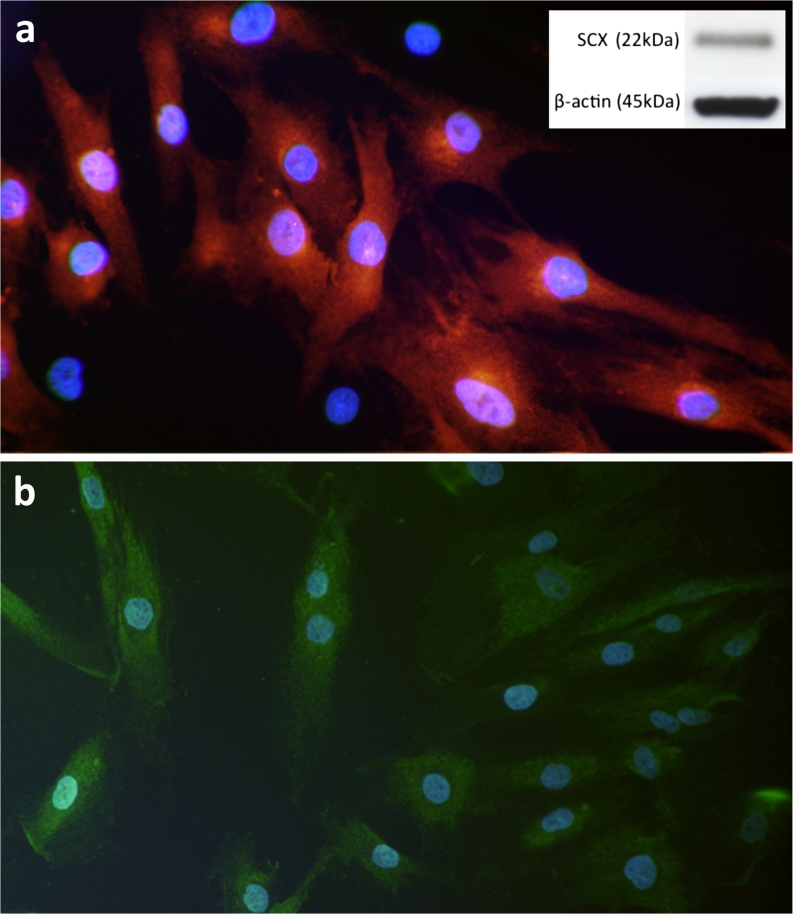
Immunocytochemical staining of primary cultures of human Achilles tendon cells. Nuclei are stained with 4,6-diamidino-2-phenylindole (DAPI; *blue*). **a** Most cells are positive for scleraxis (*red*, tetramethylrhodamine isothiocyanate [TRITC]), a key regulator of tenocyte differentiation. *Inset* Western blot showing a positive band for scleraxis corresponding to the predicted size of 22 kDa; β-actin is shown as a reference. **b** Cells showing immunoreaction for tenomodulin (*green*, fluorescein isothiocyanate [FITC]), a glycoprotein predominantly expressed in tendons and ligaments. x235 (a) and x185 (b)

**Fig. 2:**
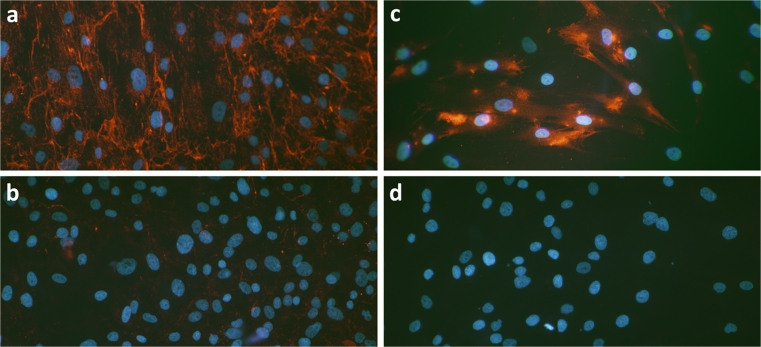
Immunocytochemical staining (*red*, TRITC) of primary cultures of human Achilles tendon cells with surrounding extracellular matrix components. Immunofluorescence for collagen I (**a**) shows that this protein is abundantly expressed in the cultures, whereas, as expected, collagen III (**b**) is only sparsely seen. The cells additionally show positive immunoreaction for connexin 43 (**c**), a gap junction protein known to be present in networks of tendon cells in vivo. The cells are negatively stained for alpha-smooth muscle actin (**d**). Nuclei are stained with DAPI (*blue*). ×120

The vast majority of cells in the primary cultures were immunopositive for vimentin, scleraxis (Fig. 1a) and tenomodulin (Fig. 1b) in the passages and serum concentrations used for experiments, thereby indicating a fibroblastic phenotype (tenocytes), as previously described for this model (Backman et al. 2011). The presence of scleraxis protein was also confirmed by using Western blot (Fig. 1a, inset) and the presence of scleraxis mRNA was confirmed by qPCR. Production of collagen I and also an extremely low expression of collagen III were further found (Fig. 2a, b). In addition, cells with myofibroblastic characteristics could also be excluded, as all cultured cells were negative for alpha-smooth muscle actin staining in the passages and serum concentrations used for the experiments (Fig. 2d). Cells were positively immunostained for connexin 43 (Fig. 2c). Connexin 43 is the main gap junction protein expected to be found in tendon cells and is known to create functional linkages among networks of tendon cells in vivo (McNeilly et al. 1996).

### ChAT, VAChT and muscarinic receptor expression in cultured tendon cells

**Fig. 3:**
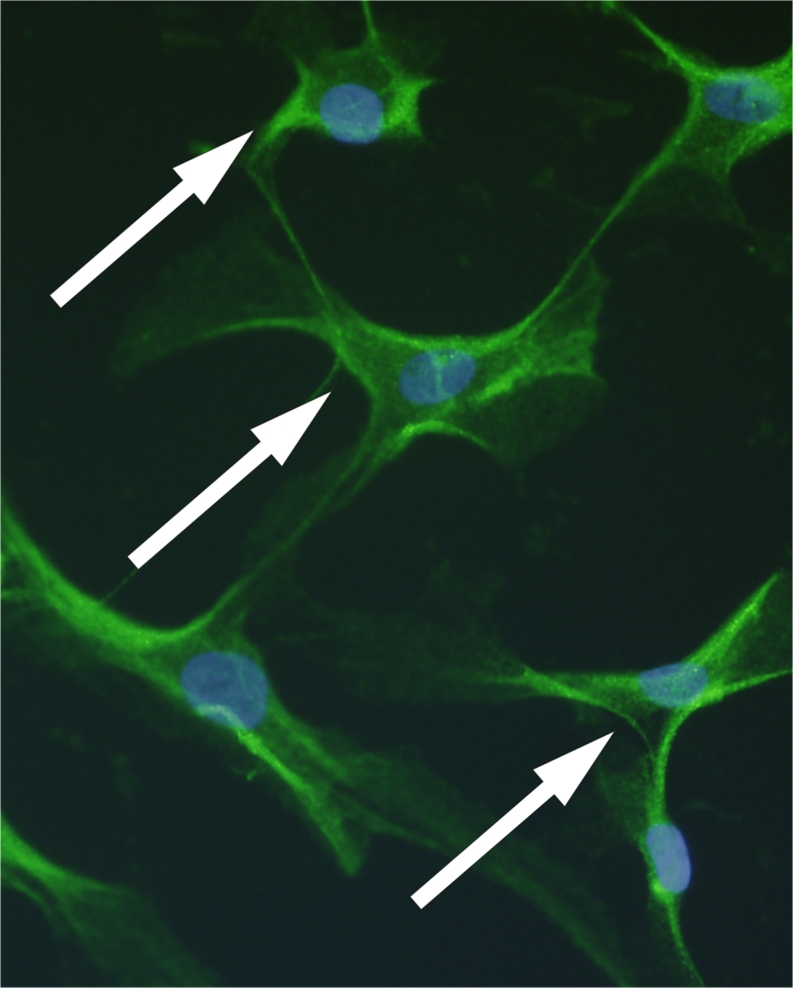
Cultured human Achilles tendon cells immunopositive (*green*, FITC; some marked with *arrows*) for the acetylcholine (ACh)-synthesising enzyme choline acetyltransferase (ChAT). DAPI staining demonstrates nuclei (*blue*). ×390

**Fig. 4:**
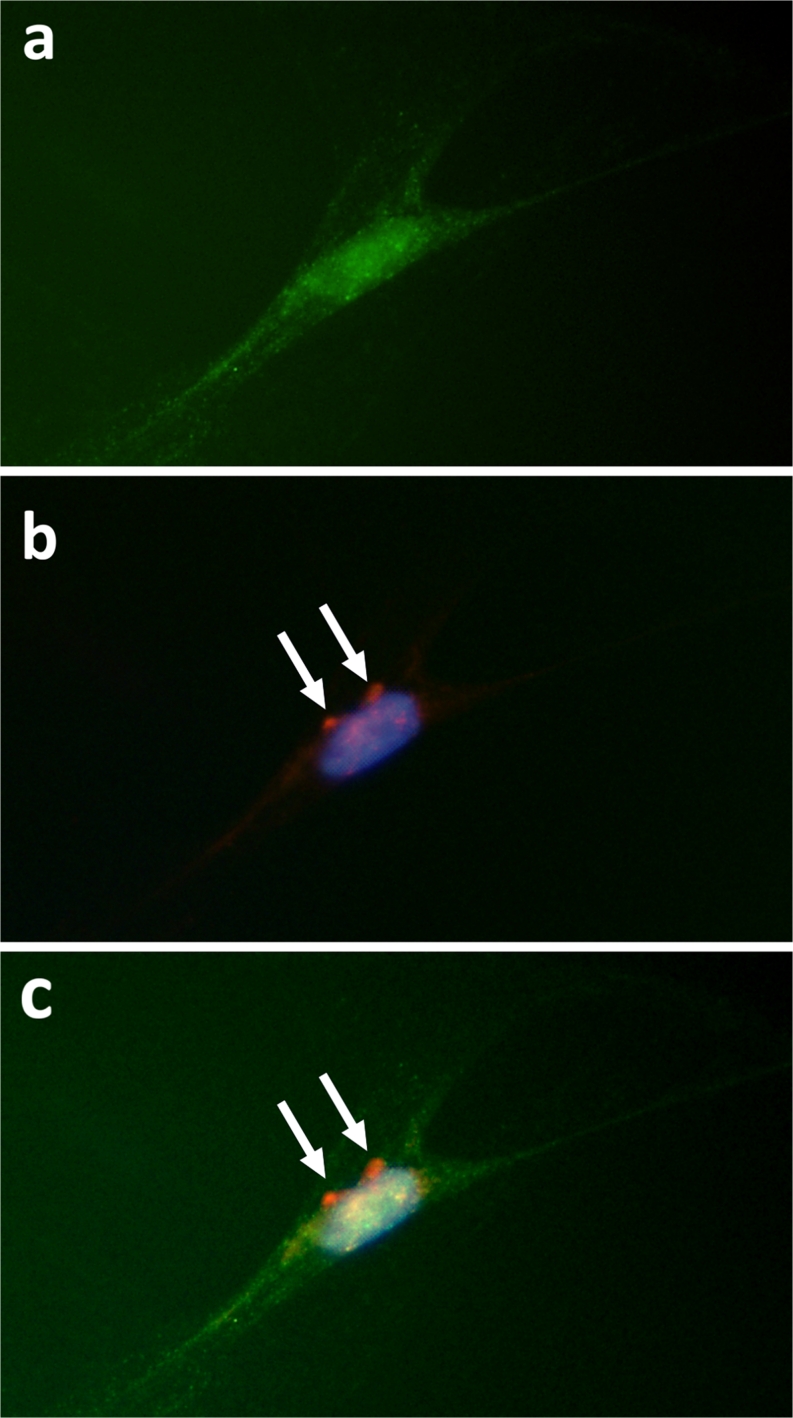
Cultured human Achilles tenocyte visualised by using immunocytochemistry. **a** The cell is immunopositive for the ACh-synthesising enzyme ChAT (*green*, FITC). **b** The same cell stained with DAPI to demonstrate the nucleus (*blue*) and immunolabelled for M_2_R (*red*, TRITC; *arrows*). **c** Digitally merged image of **a** and **b**. ×570

Most of the cultured human tendon cells were found to express clear immunoreactivity for ChAT (Figs. 3, 4a) and VAChT. For the muscarinic receptors, positive immunoreaction on cells was seen for all the studied receptors, including, as previously reported for tenocytes in tissue sections (Danielson et al. 2006, 2007; Bjur et al. 2008), M_2_R (Fig. 4b) and, in particular, M_4_R, which was expressed in a majority of the cells. In many cases, the same cells expressed both enzymes related to the synthesis/release of ACh and the muscarinic receptors (e.g. Fig. 4c). The presence of M_4_R on human tenocytes in tissue sections was confirmed by immunohistochemistry.

**Fig. 5:**
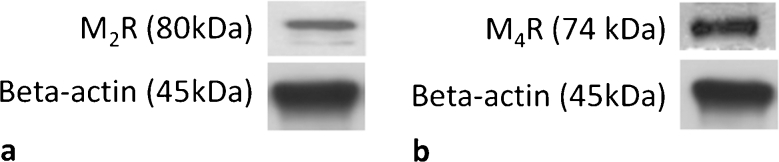
Western blots for M_2_R (**a**) and M_4_R (**b**) on cultured and lysed human primary Achilles tendon cells. Immunopositive bands are present at the expected molecular weights. β-Actin is shown as a reference.

Given earlier results that M_2_R expression was found in human tenocytes in tissue sections (Danielson et al. 2006, 2007; Bjur et al. 2008) and that M_4_R was the subtype with the highest expression in tendon cell cultures, Western blots were performed for these two subtypes to confirm their presence. Western blots for M_2_R displayed one major band at 80 kDa in lysates of cultured tendon cells (Fig. 5a). These results were in accordance with results from a positive control of purified M_2_R protein (not shown). Western blots for M_4_R displayed a band at 74 kDa corresponding to the expected size (Fig. 5b).

**Fig. 6:**
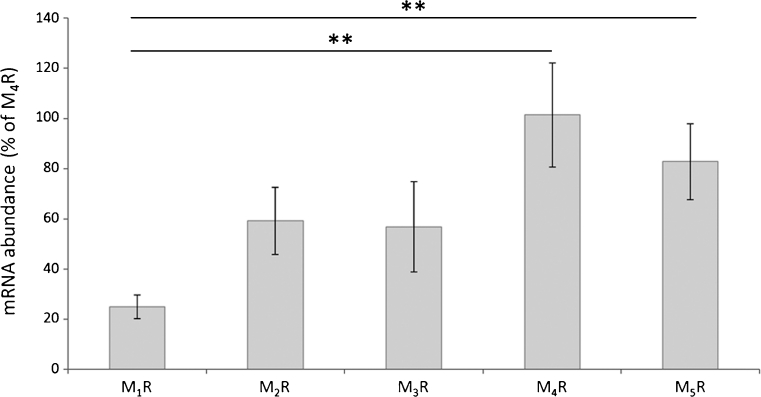
Quantitative polymerase chain reaction (qPCR) was used to study the presence of mRNA for various muscarinic receptor subtypes. The abundance of mRNA for the various subtypes was compared with that of M_4_R, which was set to 100%. The results show that mRNA for all the studied subtypes is present. The expression of M_1_R mRNA is significantly lower than that of M_4_R and M_5_R mRNA. The differences in expression between M_2_R and M_4_R mRNA (*P*=0.07) and between M_3_R and M_4_R mRNA (*P*=0.05) were close to significant (*error bars* standard deviation). **P≤0.01. One-way analysis of variance (ANOVA) with the Bonferroni post hoc test, based on triplicates in the experiment

Expression of M_1_R, M_2_R, M_3_R, M_4_R and M_5_R mRNA was also confirmed to be present in the cells by qPCR (Fig. 6). M_1_R mRNA was expressed at a significantly lower level than both M_4_R (*P*≤0.01) and M_5_R (*P*≤0.01) mRNA. A trend for the higher expression of M_4_R mRNA was also noted in comparison with both M_2_R (*P*=0.07) and M_3_R (*P*=0.05) mRNA.

### Effects of ACh

**Fig. 7:**
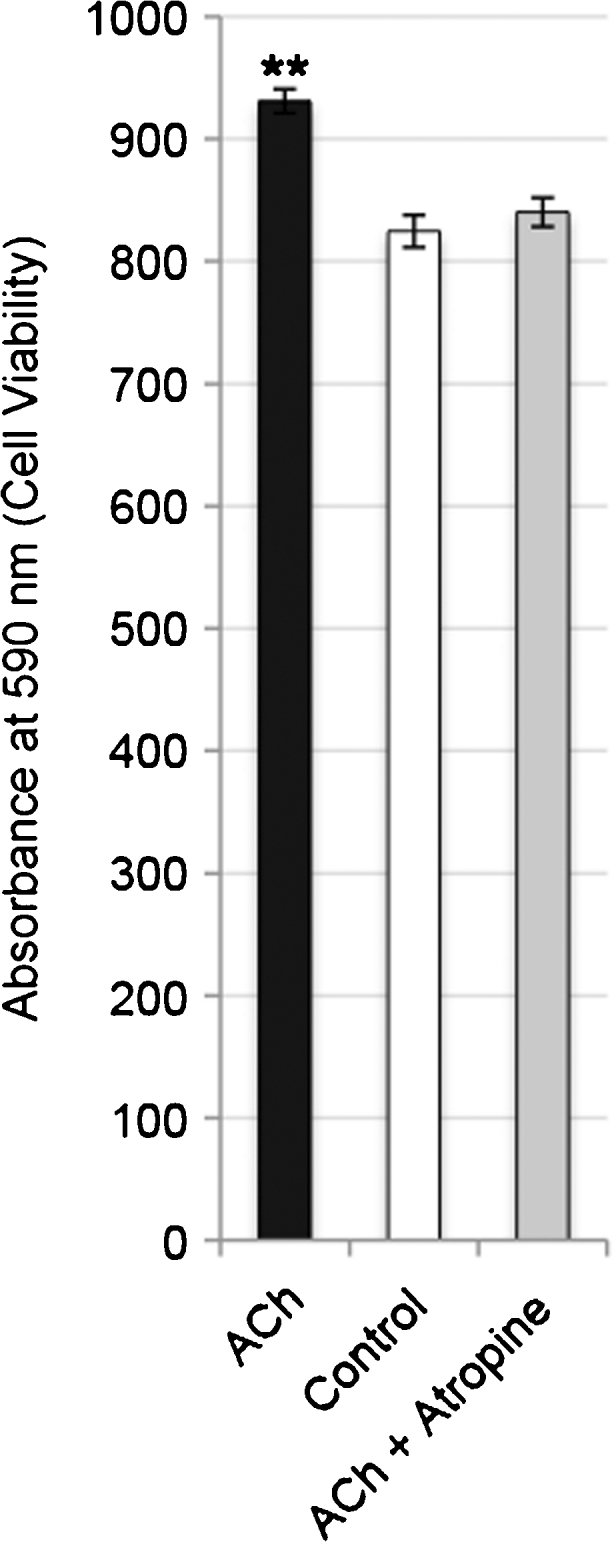
Analysis of viable tendon cells in human primary cultures after 36 h of incubation with 10^−6^ M acetylcholine (*ACh*), without ACh (*Control*) and with ACh and the muscarinic ACh receptor blocker atropine (*ACh + Atropine*; the concentration of atropine being 10^−5^ M) measured by absorbance (590 nm) of crystal violet staining. The significant increase in viable cells seen after incubation with ACh is effectively blocked with atropine. Cells were seeded at a density of 1.5×10^5^ cells/well in 6-well plates (*error bars* standard deviation). **P<0.01 for ACh against both Control and ACh + Atropine. One-way ANOVA with the Bonferroni post hoc test, based on triplicates in the experiment

Exogenously administered ACh significantly increased the number of viable tendon cells after 36 h of incubation as seen by crystal violet staining (*P*<0.01; one-way ANOVA with the Bonferroni post-hoc test) and this effect was effectively blocked by simultaneous incubation with the muscarinic ACh receptor antagonist atropine (Fig. 7).

**Fig. 8:**
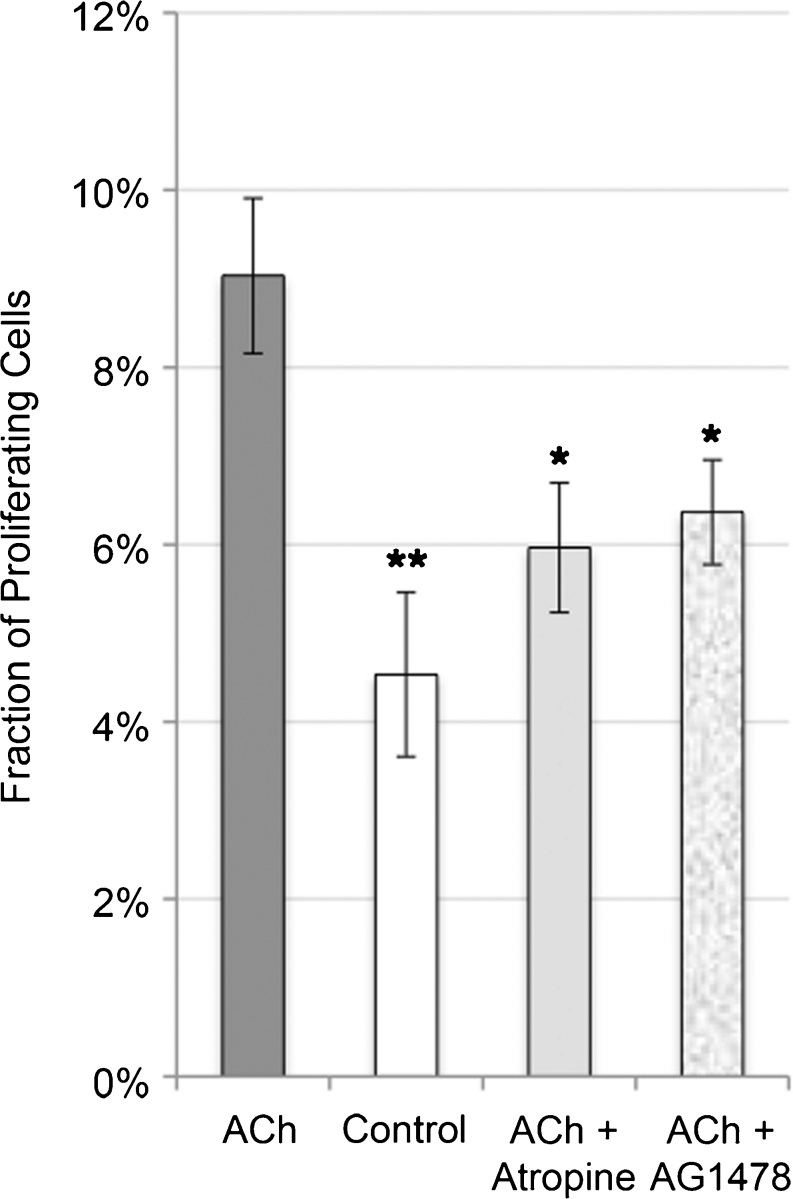
Mean fraction of proliferating (BrdU-positive) tendon cells in human primary cultures after 24 h of incubation with ACh (10^−6^ M), without ACh (*Control*), with ACh and the muscarinic receptor inhibitor atropine (10^−5^ M) and with ACh and the epidermal growth factor receptor (EGFR) inhibitor AG1478 (0.8 μM). A statistically significant increase can be seen in the percentage of proliferating cells after incubation with ACh compared with the control (**P<0.01) and compared with incubation with the inhibitors (*P<0.05). No significant difference can be found between the treatments with atropine and AG1478 or between either of the inhibitors and the control. Cells were seeded at a density of 1.5×10^4^ cells/well in 8-well chamber slides (*error bars* standard deviation). One-way ANOVA test with the Bonferroni post hoc test, based on triplicates in the experiment

The percentage of proliferating (BrdU-positive) tendon cells in cultures after incubation with ACh was significantly increased (doubled) after 24 h compared with the controls (*P*<0.01) but also with cells incubated with atropine (*P*<0.05) or the EGFR-blocker AG1478 (*P*<0.05; Fig. 8; one-way ANOVA with the Bonferroni post-hoc test).

**Fig. 9:**
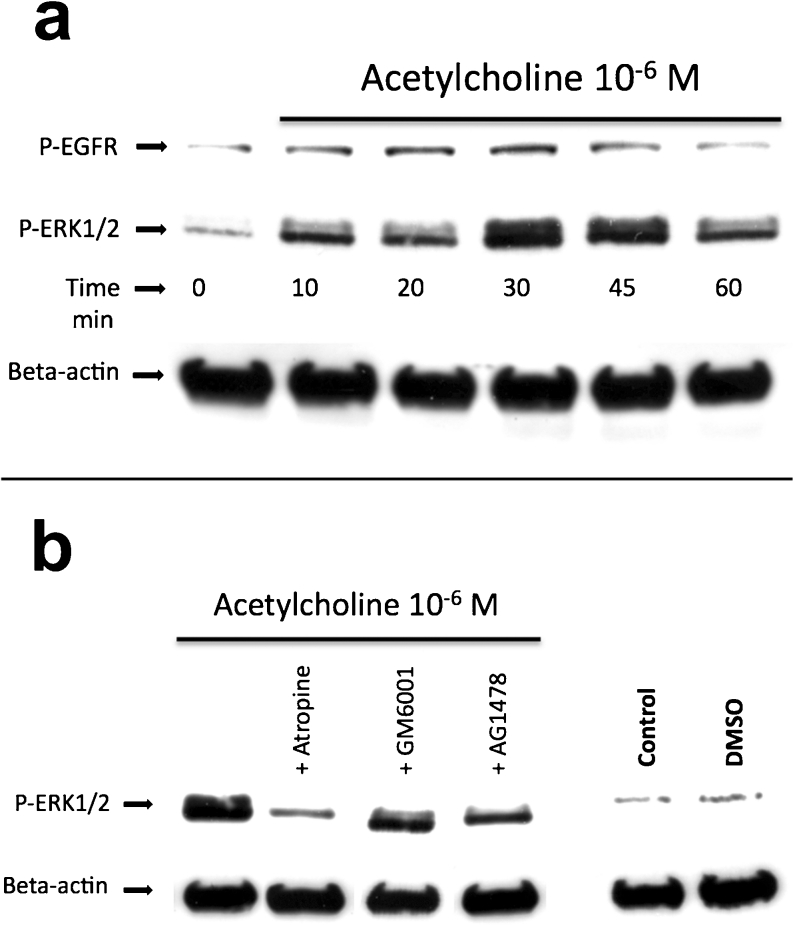
**a** Western blot showing phosphorylated EGFR (*P-EGFR*) and extracellular-signal-regulated kinases 1 and 2 (*P-ERK1/2*) in cultured human Achilles tendon cells at various time points after incubation with ACh (10^−6^ M). Exposure to ACh resulted in phosphorylation (i.e. activation) of both EGFR and ERK1/2 in the cultured cells; this activation peaked after 20–30 min for EGFR and 30–45 min for ERK1/2. β-Actin is shown as a reference. **b** Western blot showing phosphorylated ERK1/2 (*P-ERK1/2*) in cultured human Achilles tendon cells after 45 min of incubation in pure ACh (10^−6^ M), ACh and the muscarinic ACh receptor antagonist atropine (*+Atropine*), ACh and a metalloproteinase (MMP) antagonist (*+GM6001*), ACh and an EGFR antagonist (*+AG1478*), regular media (*Control*) or dimethylsuphoxide (*DMSO*). ERK1/2 activation by ACh is effectively blocked when incubated simultaneously with atropine. Moreover, inhibition of either EGFR or MMP reduces the phosphorylation of ERK1/2. β-Actin is shown as a reference

Western blot analysis revealed that the administration of ACh resulted in the phosphorylation (i.e. activation) of both EGFR and ERK1/2 in the cultured cells. This activation peaked after 20–30 min for EGFR and after 30–45 min for ERK1/2 (Fig. 9a).

The ERK1/2 phosphorylation induced by incubation with ACh was effectively blocked in the presence of the muscarinic receptor antagonist, atropine (Fig. 9b). In addition, inhibition of either EGFR or MMP with their specific blockers (AG1478 and GM6001, respectively) decreased the phosphorylation of ERK1/2 (Fig. 9b).

## Discussion

The present study shows that human Achilles tenocytes express enzymes related to ACh synthesis/release (ChAT and VAChT) and mAChRs in vitro, findings previously shown for these cells in vivo, especially in tendinosis (Danielson et al. 2006, 2007; Bjur et al. 2008). Furthermore, we demonstrate that these cells are encouraged, when exposed to ACh, to proliferate via mAChR stimulation and subsequently the activation of EGFR and ERK1/2.

Previous reports have shown that the effects of mAChR activation include fibroblast proliferation (Matthiesen et al. 2006; Profita et al. 2009), mediated through the activation of the ERK1/2 pathway (Matthiesen et al. 2007). Other studies have shown that the stimulation of these receptors on fibroblasts increases collagen synthesis in the lungs (Haag et al. 2008) as a part of tissue remodelling in response to chronic inflammation (Jeffery 2001). In human Achilles tenocytes, we have, in the present study, observed that, after 24 h, the number of cells in the S-phase of the cell cycle (BrdU-positive) is significantly higher in the ACh-exposed cells and also that, after an additional 12 h, the number of viable cells is significantly increased as a result. Collagen synthesis has not been quantified in this study but this would surely be an interesting next step, as tendinosis is characterised by increased collagen type III content, which is also seen in the early stages of tendon healing (Maffulli et al. 2000).

**Fig. 10:**
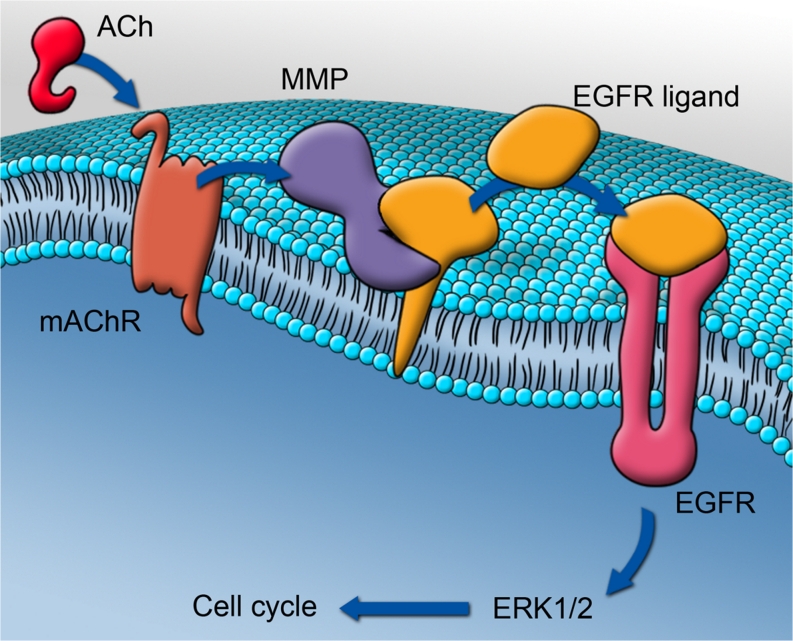
Representation of the proposed cellular pathways involved in the acetylcholine (*ACh*)-induced proliferation of human tendon cells. ACh stimulates membrane-bound muscarinic ACh receptors (*mAChR*) that in turn activate matrix metalloproteinases (*MMP*) to cleave a cell-surface-associated epidermal growth factor receptor ligand (*EGFR ligand*). The ligand then binds to EGFR, which in turn increases cell proliferation through the phosphorylation of the mitogen-activated protein kinases, extracellular-signal-regulated kinases 1 and 2 (*ERK1/2*). The results of the present study identify a potential role for each of these steps (see Figs. 8, 9). Artwork by G. Andersson

The cultured tendon cells of the present model have been shown to retain a fibroblastic phenotype in the early passages used for the experiments here, by the immunofluorescence for vimentin (typical of mesechyme-derived cells, such as fibroblasts; Rufai et al. 1992) and tenomodulin (a specific marker for tenocytes; Docheva et al. 2005; Jelinsky et al. 2010), a finding confirmed in an earlier study of our model (Backman et al. 2011). Tenomodulin is a type II transmembrane glycoprotein that is highly expressed in tendons and ligaments and it has been reported to be positively regulated by scleraxis, which is a transcription factor that also regulates the expression of type I collagen, thus being a key transcription factor in tenocyte differentiation (Shukunami et al. 2006; Lejard et al. 2007; Jelinsky et al. 2010). Scleraxis has been observed at both the protein and the mRNA levels in the cells of this study. The proliferative effect of ACh on these fibroblastic cells has been found to be mAChR-dependent and to lead to EGFR and ERK1/2 activation. An overview of the proposed pathway is presented in Fig. 10.

The specific MMP responsible for cleaving the pro-ligand to a mature EGFR ligand has not been defined in this study. However, we have observed a tendency towards decreased ERK1/2 phosphorylation in response to the broad-spectrum MMP inhibitor GM6001, which has MMP-1 as its primary target and MMP-2, −3, −8 and −9 as secondary targets. The finding that the inhibitory effect is not more dramatic might be attributable to the involvement of other MMPs that are not affected by GM6001. For example, in colon cancer cells, Xie et al. (2009) have shown that ACh induces proliferation involving MMP-1, MMP-7 and MMP-10, in turn, leading to EGFR activation, which can be abolished with the muscarinic inhibitor atropine and that the stimulation of MMP-7 mimics the actions of ACh. Another possible agent is the tumor necrosis factor alpha (TNF_α_)-converting enzyme (TACE or ADAM17). This enzyme is known to cleave TNF_α_ but has also been found to be the only metalloprotinease that efficiently cleaves transforming growth factor-alpha (TGF_α_), another EGFR ligand (Hinkle et al. 2003). GPCR stimulation in colonocytes leads to the cleavage and release of the mature TGF_α_, which binds to, and phosphorylates, EGFR (Koon et al. 2004).

The targeting of mAChRs is frequently used in the clinical setting to abolish unwanted effects of ACh and mAChRs have been proposed as potential targets in several diseases, such as chronic obstructive pulmonary disease in which the inhibition of mAChRs can delay the decline in lung function (Vincken et al. 2002; Anzueto et al. 2005). The mAChR family can be divided into the “odd-numbered” and the “even-numbered” receptors. The “even-numbered” receptors M_2_R and M_4_R couple via G_i_ and G_o α_ subunits (Caulfield and Birdsall 1998). In lung fibroblasts, M_2_R has been found to be the most prominent mAChR and the regulation of fibroblast proliferative rate has been shown to correlate best with modulation via M_2_R (Matthiesen et al. 2006). In this study, we have confirmed the presence of M_2_R protein and mRNA in cultured human tenocytes, findings previously shown for tenocytes in tissue sections from both patellar (Danielson et al. 2006) and Achilles (Bjur et al. 2008) tendons in man. However, the expression of the other known mAChRs has not previously been examined in human tendons. In this study, we have also investigated M_3_R, M_4_R and M_5_R, with M_4_R clearly being the most abundantly expressed receptor in the in vitro setting. The expression of M_4_R has, in the present study, also been confirmed for tenocytes in tissue sections. This study has not investigated the possible expression or role of nicotinic ACh receptors, which is a drawback. Nevertheless, since the proliferative effect of ACh in this study is abolished with atropine, we have confirmed that proliferation takes place preferably through muscarinic stimulation and not nicotinic receptor stimulation.

The verification of ACh production by human tenocytes is based on the expression of ChAT and VAChT and not on a measurement of the ACh molecule itself. This is of course a limitation of this study, although, as has been repeatedly reported, the expression of ChAT and VAChT is correlated to ACh production. ChAT catalyses the synthesis of ACh from choline and acetyl-CoA (Tuček 1982, 1988) and VAChT is known then to shuttle ACh to vesicles in the nerve terminal (Eiden 1998). Over-expression of VAChT is related to an increased ACh release (Prado et al. 2002). All in all, the present study supports the existence of a cholinergic autocrine loop in tenocytes (Forsgren et al. 2009a, 2009b), ACh produced by the cells themselves being able to stimulate the proliferation of the same cells through the activation of the ERK1/2 system via binding to the mAChRs on the cell surface. The potential relevance of this phenomenon to tendinosis is evident, given that hypercellularity is such a prominent feature in the histopathology, both at early and late stages (Khan et al. 1999). Nevertheless, other possible targets of the ACh produced by tenocytes in tendon tissue might be receptors found in blood vessel walls or on sensory nerve fascicles (Danielson et al. 2006; Bjur et al. 2008). These receptor sites are also of interest, since ACh has well-known vascular and pain-modulating effects (Vogelsang et al. 1995; Dussor et al. 2004), tendinosis also being characterised by vascular changes and chronic pain (Khan et al. 1999; Alfredson and Ohberg 2005; Cook et al. 2005).

In summary, based on the results of the present study and on those of our previous investigations (Danielson et al. 2006, 2007; Bjur et al. 2008), we propose the following. Human tenocytes, harbouring the capability to produce ACh, increase this production during tendinosis development. Via an autocrine loop, the ACh produced by tenocytes stimulates mAChRs on the cell surface, which in their turn increase ERK1/2 phosphorylation through the activation of EGFR and contribute to the increased cell proliferation (Fig. 10) and the tenocyte hypercellularity seen in tendinosis. In an early stage of tendinosis, hypercellularity might be a part of a healing or adaptive response but, in the chronic stage, excessive tenocyte proliferation could be detrimental to tendon structure and function. The non-neuronal cholinergic system of tendon tissue is thus a possible target for future modulation of these processes in tendinosis.

## References

[CR1] Alfredson H, Ohberg L (2005). Neovascularisation in chronic painful patellar tendinosis—promising results after sclerosing neovessels outside the tendon challenge the need for surgery. Knee Surg Sports Traumatol Arthrosc.

[CR2] Anzueto A, Tashkin D, Menjoge S, Kesten S (2005). One-year analysis of longitudinal changes in spirometry in patients with COPD receiving tiotropium. Pulm Pharmacol Ther.

[CR3] Backman LJ, Fong G, Andersson G, Scott A, Danielson P (2011). Substance P is a mechanoresponsive, autocrine regulator of human tenocyte proliferation. PLoS One.

[CR4] Bjur D, Danielson P, Alfredson H, Forsgren S (2008) Presence of a non-neuronal cholinergic system and occurrence of up- and down-regulation in expression of M2 muscarinic acetylcholine receptors: new aspects of importance regarding Achilles tendon tendinosis (tendinopathy). Cell Tissue Res 331:385–400 10.1007/s00441-007-0524-1 17999088

[CR5] Caulfield MP, Birdsall NJ (1998). International union of pharmacology. XVII. Classification of muscarinic acetylcholine receptors. Pharmacol Rev.

[CR6] Cook JL, Malliaras P, De Luca J, Ptasznik R, Morris M (2005). Vascularity and pain in the patellar tendon of adult jumping athletes: a 5 month longitudinal study. Br J Sports Med.

[CR7] Danielson P (2009). Reviving the “biochemical” hypothesis for tendinopathy: new findings suggest the involvement of locally produced signal substances. Br J Sports Med.

[CR8] Danielson P, Alfredson H, Forsgren S (2006). Immunohistochemical and histochemical findings favoring the occurrence of autocrine/paracrine as well as nerve-related cholinergic effects in chronic painful patellar tendon tendinosis. Microsc Res Tech.

[CR9] Danielson P, Andersson G, Alfredson H, Forsgren S (2007). Extensive expression of markers for acetylcholine synthesis and of M2 receptors in tenocytes in therapy-resistant chronic painful patellar tendon tendinosis—a pilot study. Life Sci.

[CR10] Docheva D, Hunziker EB, Fassler R, Brandau O (2005). Tenomodulin is necessary for tenocyte proliferation and tendon maturation. Mol Cell Biol.

[CR11] Dussor GO, Helesic G, Hargreaves KM, Flores CM (2004). Cholinergic modulation of nociceptive responses in vivo and neuropeptide release in vitro at the level of the primary sensory neuron. Pain.

[CR12] Eiden LE (1998). The cholinergic gene locus. J Neurochem.

[CR13] Forsgren S, Alfredson H, Bjur D, Rantapaa-Dahlqvist S, Norrgard O, Dalen T, Danielson P (2009). Novel information on the non-neuronal cholinergic system in orthopedics provides new possible treatment strategies for inflammatory and degenerative diseases. Orthop Rev (Pavia).

[CR14] Forsgren S, Grimsholm O, Jonsson M, Alfredson H, Danielson P (2009). New insight into the non-neuronal cholinergic system via studies on chronically painful tendons and inflammatory situations. Life Sci.

[CR15] Haag S, Matthiesen S, Juergens UR, Racke K (2008). Muscarinic receptors mediate stimulation of collagen synthesis in human lung fibroblasts. Eur Respir J.

[CR16] Hinkle CL, Mohan MJ, Lin P, Yeung N, Rasmussen F, Milla ME, Moss ML (2003). Multiple metalloproteinases process protransforming growth factor-alpha (proTGF-alpha). Biochem.

[CR17] Jacobi J, Jang JJ, Sundram U, Dayoub H, Fajardo LF, Cooke JP (2002). Nicotine accelerates angiogenesis and wound healing in genetically diabetic mice. Am J Pathol.

[CR18] Jeffery PK (2001). Remodeling in asthma and chronic obstructive lung disease. Am J Respir Crit Care Med.

[CR19] Jelinsky SA, Archambault J, Li L, Seeherman H (2010). Tendon-selective genes identified from rat and human musculoskeletal tissues. J Orthop Res.

[CR20] Kawashima K, Fujii T (2003). The lymphocytic cholinergic system and its contribution to the regulation of immune activity. Life Sci.

[CR21] Khan KM, Cook JL, Bonar F, Harcourt P, Astrom M (1999). Histopathology of common tendinopathies. Update and implications for clinical management. Sports Med.

[CR22] Koon HW, Zhao D, Na X, Moyer MP, Pothoulakis C (2004). Metalloproteinases and transforming growth factor-alpha mediate substance P-induced mitogen-activated protein kinase activation and proliferation in human colonocytes. J Biol Chem.

[CR23] Kraus S, Benard O, Naor Z, Seger R (2003). c-Src is activated by the epidermal growth factor receptor in a pathway that mediates JNK and ERK activation by gonadotropin-releasing hormone in COS7 cells. J Biol Chem.

[CR24] Lejard V, Brideau G, Blais F, Salingcarnboriboon R, Wagner G, Roehrl MH, Noda M, Duprez D, Houillier P, Rossert J (2007). Scleraxis and NFATc regulate the expression of the pro-alpha1(I) collagen gene in tendon fibroblasts. J Biol Chem.

[CR25] Maffulli N, Ewen SW, Waterston SW, Reaper J, Barrass V (2000). Tenocytes from ruptured and tendinopathic Achilles tendons produce greater quantities of type III collagen than tenocytes from normal Achilles tendons. An in vitro model of human tendon healing. Am J Sports Med.

[CR26] Matthiesen S, Bahulayan A, Kempkens S, Haag S, Fuhrmann M, Stichnote C, Juergens UR, Racke K (2006). Muscarinic receptors mediate stimulation of human lung fibroblast proliferation. Am J Respir Cell Mol Biol.

[CR27] Matthiesen S, Bahulayan A, Holz O, Racke K (2007). MAPK pathway mediates muscarinic receptor-induced human lung fibroblast proliferation. Life Sci.

[CR28] McNeilly CM, Banes AJ, Benjamin M, Ralphs JR (1996). Tendon cells in vivo form a three dimensional network of cell processes linked by gap junctions. J Anat.

[CR29] New DC, Wong YH (2007). Molecular mechanisms mediating the G protein-coupled receptor regulation of cell cycle progression. J Mol Signal.

[CR30] Oben JA, Yang S, Lin H, Ono M, Diehl AM (2003). Acetylcholine promotes the proliferation and collagen gene expression of myofibroblastic hepatic stellate cells. Biochem Biophys Res Commun.

[CR31] Prado MA, Reis RA, Prado VF, Mello MC de, Gomez MV, Mello FG de (2002) Regulation of acetylcholine synthesis and storage. Neurochem Int 41:291–299 10.1016/s0197-0186(02)00044-x 12176069

[CR32] Profita M, Bonanno A, Siena L, Bruno A, Ferraro M, Montalbano AM, Albano GD, Riccobono L, Casarosa P, Pieper MP, Gjomarkaj M (2009). Smoke, choline acetyltransferase, muscarinic receptors, and fibroblast proliferation in chronic obstructive pulmonary disease. J Pharmacol Exp Ther.

[CR33] Rozengurt E (2007). Mitogenic signaling pathways induced by G protein-coupled receptors. J Cell Physiol.

[CR34] Rufai A, Benjamin M, Ralphs JR (1992). Development and ageing of phenotypically distinct fibrocartilages associated with the rat Achilles tendon. Anat Embryol.

[CR35] Shukunami C, Takimoto A, Oro M, Hiraki Y (2006). Scleraxis positively regulates the expression of tenomodulin, a differentiation marker of tenocytes. Dev Biol.

[CR36] Tuček S (1982). The synthesis of acetylcholine in skeletal muscles of the rat. J Physiol (Lond).

[CR37] Tuček S, Whittaker VP (1988). Choline acetyltransferase and the synthesis of acetylcholine. Handbook of experimental pharmacology, vol 86. The cholinergic synapse.

[CR38] Vincken W, Noord JA van, Greefhorst AP, Bantje TA, Kesten S, Korducki L, Cornelissen PJ (2002) Improved health outcomes in patients with COPD during 1 year’s treatment with tiotropium. Eur Respir J 19:209–216 10.1183/09031936.02.00238702 11871363

[CR39] Vogelsang M, Heyer G, Hornstein OP (1995). Acetylcholine induces different cutaneous sensations in atopic and non-atopic subjects. Acta Derm Venereol.

[CR40] Wess J, Duttaroy A, Gomeza J, Zhang W, Yamada M, Felder CC, Bernardini N, Reeh PW (2003). Muscarinic receptor subtypes mediating central and peripheral antinociception studied with muscarinic receptor knockout mice: a review. Life Sci.

[CR41] Wessler I, Kirkpatrick CJ, Racke K (1998). Non-neuronal acetylcholine, a locally acting molecule, widely distributed in biological systems: expression and function in humans. Pharmacol Ther.

[CR42] Wessler I, Kilbinger H, Bittinger F, Kirkpatrick CJ (2001). The biological role of non-neuronal acetylcholine in plants and humans. Jpn J Pharmacol.

[CR43] Xie G, Cheng K, Shant J, Raufman JP (2009). Acetylcholine-induced activation of M3 muscarinic receptors stimulates robust matrix metalloproteinase gene expression in human colon cancer cells. Am J Physiol Gastrointest Liver Physiol.

